# Epistatic Interactions in the Arabinose *Cis*-Regulatory Element

**DOI:** 10.1093/molbev/msv269

**Published:** 2015-11-20

**Authors:** Mato Lagator, Claudia Igler, Anaísa B. Moreno, Călin C. Guet, Jonathan P. Bollback

**Affiliations:** ^1^IST Austria, Klosterneuburg, Austria

**Keywords:** gene expression, gene regulation, epistasis, *cis*-regulatory elements

## Abstract

Changes in gene expression are an important mode of evolution; however, the proximate mechanism of these changes is poorly understood. In particular, little is known about the effects of mutations within *cis* binding sites for transcription factors, or the nature of epistatic interactions between these mutations. Here, we tested the effects of single and double mutants in two *cis* binding sites involved in the transcriptional regulation of the *Escherichia coli araBAD* operon, a component of arabinose metabolism, using a synthetic system. This system decouples transcriptional control from any posttranslational effects on fitness, allowing a precise estimate of the effect of single and double mutations, and hence epistasis, on gene expression. We found that epistatic interactions between mutations in the *araBAD cis-*regulatory element are common, and that the predominant form of epistasis is negative. The magnitude of the interactions depended on whether the mutations are located in the same or in different operator sites. Importantly, these epistatic interactions were dependent on the presence of arabinose, a native inducer of the *araBAD* operon in vivo, with some interactions changing in sign (e.g., from negative to positive) in its presence. This study thus reveals that mutations in even relatively simple *cis-*regulatory elements interact in complex ways such that selection on the level of gene expression in one environment might perturb regulation in the other environment in an unpredictable and uncorrelated manner.

## Introduction

Changes in the *cis* regulation of gene expression have been proposed as a major source of evolutionary innovation (King and Wilson 1975; Wittkopp and Kalay 2012). For example, across insect species there has been increasing evidence for the essential role that *cis* regulatory changes have in shaping body plan formation (Carroll 2000, 2008; Wittkopp and Kalay 2012). Changes in the regulation of gene expression can occur through mutations in the transcription factor coding sequence (transregulatory elements) and/or in *cis*-regulatory elements (CREs), which contain the transcription factor and the RNA polymerase (RNAP) binding sites (Jacob and Monod 1961). Mutations in CREs may be important targets of selection (Stern and Orgogozo 2008), as it is hypothesized that, compared with *trans* elements, mutations in CREs have a wider range of effects, giving rise to a greater diversity of phenotypes that could be selected upon (Wray 2007).

Previous studies have described distributions of mutational effects for several prokaryotic CREs both in vitro (Maerkl and Quake 2007, 2009; Geertz et al. 2012) and in vivo (Patwardhan et al. 2009; Kinney et al. 2010; Brewster et al. 2012; Sharon et al. 2012; Kosuri et al. 2013). As these studies predominantly focused on characterizing general relationships between *trans* factors (TFs and RNAP) and their *cis* binding sites, the analysis of interactions between individual *cis* mutations has been limited (Kwasnieski et al. 2012), partly due to restrictions in the techniques used (Melnikov et al. 2012; Patwardhan et al. 2012). Understanding the dependence of the effect of a mutation on the genetic background in which it appears, a phenomenon termed epistasis (Fisher 1918; Phillips 2008) is critical to understanding adaptation and the engineering of synthetic promoters with specific properties (Kinkhabwala and Guet 2008). That is, the phenotypes (gene expression levels, in the case of CREs) of individuals containing multiple mutations are expected to correspond to those of the underlying single mutant phenotypes. Instead, however, the phenotypes might deviate from this expectation, resulting in positive epistasis if the mean double mutant expression is greater than the expression level predicted from single mutants. If the double mutant expression is lower than predicted, then mutations are considered to be in negative epistasis. If the individual mutation causes an increase in the expression but the double mutant containing that mutation leads to its reduction (or vice versa), the mutation is deemed to be in sign epistasis (Phillips 2008). Epistatic interactions between mutations in CREs define the robustness as well as the evolvability of regulatory elements—not only how transcription levels can be modulated but also how new functional CREs could evolve (Payne and Wagner 2014).

**Fig. 1.**
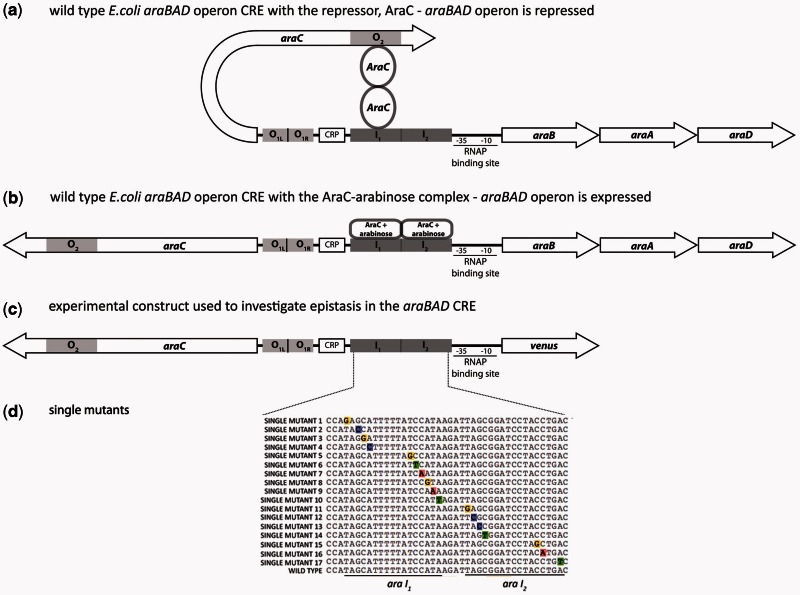
Structure of *araBAD* operon and regulatory function of AraC. The *cis-*regulatory region of the *araBAD* operon consists of two proximal AraC operators, *araI_1_* and *araI_2_*, two intermediate operators, *araO_1L_* and *araO_1R_*, and the distal operator, *araO_2_.* The *cis-*regulatory element also contains a CRP binding site. (*a*) In the absence of arabinose, AraC preferentially binds to operators *araI_1_* and *araO_2_*, forming a DNA loop and repressing transcription. (*b*) In the presence of arabinose, the AraC–arabinose complex binds to *araI_1_* and *araI_2_* operator sites, actively recruiting RNA polymerase and promoting transcription. (*c*) The structure of the experimental synthetic construct in which the *araBAD* operon has been replaced by a fluorescence reporter gene, *venus-yfp.* (*d*) We introduced point mutations in all base pairs in *araI_1_* and *araI_2_* operators for which it was previously determined through gel shift assays that any substitution decreased AraC binding by at least 10-fold (Niland et al. 1996).

The functional effects of epistatic interactions are complex and therefore poorly understood, as epistasis may be influenced by many factors (Lehner 2011). Epistasis can depend on environmental factors, as has been demonstrated for mutations in bacterial and bacteriophage proteins (You and Yin 2002; Hayden and Wagner 2012; Lalic and Elena 2012; Wang, Diaz Arenas, et al. 2012; de Vos et al. 2013; Flynn et al. 2013; Caudle et al. 2014). As cellular responses to environmental changes can be complex, understanding their effect on epistasis is inherently difficult. Some prokaryotic regulatory pathways, however, offer a tractable system for understanding the environmental response, with predictable responses to single stimuli modulating gene expression (Browning and Busby 2004). One such regulatory pathway is that of the well-studied *Escherichia coli* arabinose operon *araBAD* (Helling and Weinberg 1963; Schleif 2000) (fig. 1). This operon is activated in the presence of its substrate, the sugar arabinose, which interacts with the transcription factor AraC. By synthetically associating the *araBAD* CRE with a fluorescence marker instead of the native *araBAD* operon, we have decoupled the effects of mutations in CRE on expression from any potential downstream effects that might affect global expression (fig. 1*c*). This allows us to directly study the effects of mutations on CRE and their epistatic interactions, in the presence and absence of a single, well-understood, and controlled environmental variable, arabinose.

Epistatic interactions can also depend on the physical location of mutations. For example, the type and magnitude of interactions can be different for pairs of mutations found within a gene and those in different genes (Szendro et al. 2013), or for mutations found in genes that interact and those that do not (Lalic and Elena 2012). We investigated if the differences in epistasis in CRE might depend on whether those mutations are found in the same or in different operators. Epistasis in a CRE could arise from the effects of mutations on transcription factor binding to its operator site. But it could also arise from an interaction between the effects of mutations and the constraints imposed by protein–protein interactions that stabilize transcription factor DNA binding, as could be true for both AraC and the AraC–arabinose complex, which bind as dimers (Schleif 2003). Epistasis could also depend on the relative importance of an operator site for the control of transcription, which could be the case as both AraC and AraC–arabinose complex preferentially binding to *araI_1_* (Schleif 2010).

Our knowledge of epistasis comes almost exclusively from studying the effects of mutations on protein-coding sequences. With the exception of a few studies that looked at pairwise interactions (Kwasnieski et al. 2012), statistical epistasis (Otwinowski and Nemenman 2013), and broad patterns of interactions (Patwardhan et al. 2009, 2012; Sharon et al. 2012; Jolma et al. 2013), assessments of epistatic interactions between mutations in CREs have largely been absent. Given the potentially large role played by changes in *cis* regulatory regions on shaping evolutionary outcomes, we explored how epistatic interactions in a CRE depend on both the environment and the location of mutations. We introduced single and double point mutations into *araI_1_* and *araI_2_*, the two proximal AraC operator sites of the *araBAD* CRE, and studied the effects of these mutations on expression in two different environments, defined by the presence or absence of the natural inducer arabinose. We found that both the environment and the location of the mutations in the CRE affect the nature of epistatic interactions.

## Results

### Mutational Effects

**Fig. 2.**
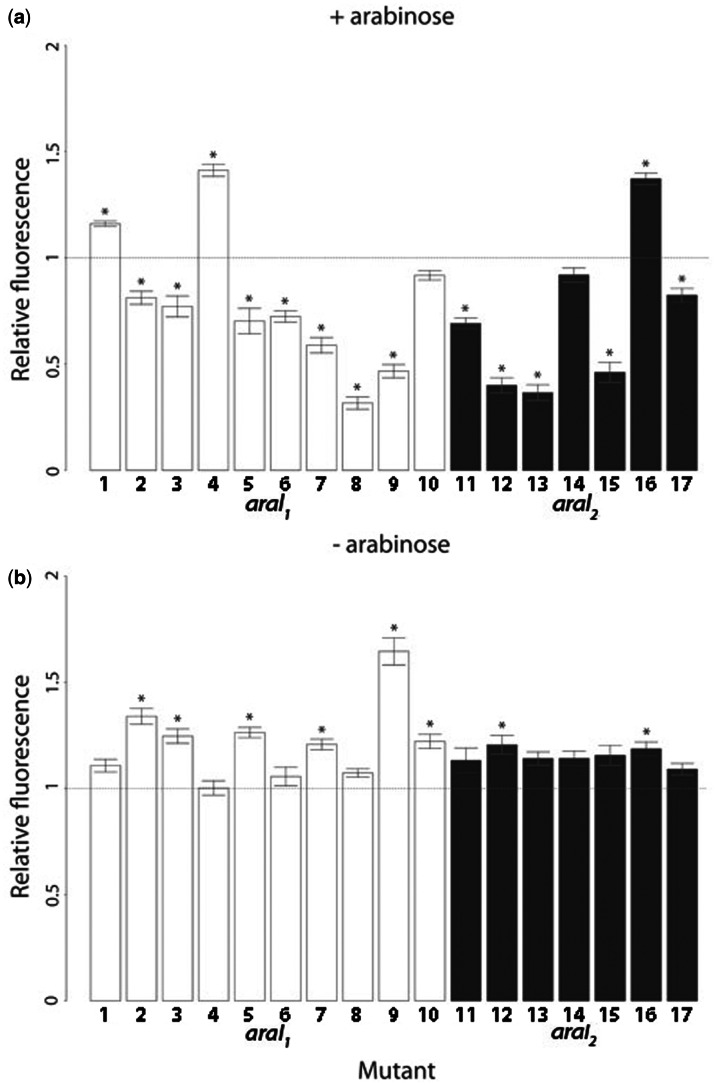
Relative fluorescence of single mutants in two environments. Bars are mean fluorescence relative to wild type. White bars are single mutants in the operator *araI*_1_, dark bars are single mutants in *araI_2_.* Stars indicate mutants that significantly differ from the wild type. The dotted line represents wild-type fluorescence, normalized to 1. Error bars are standard errors of the mean. Measurements were taken in the (*a*) presence and (*b*) absence of arabinose.

**Fig. 3.**
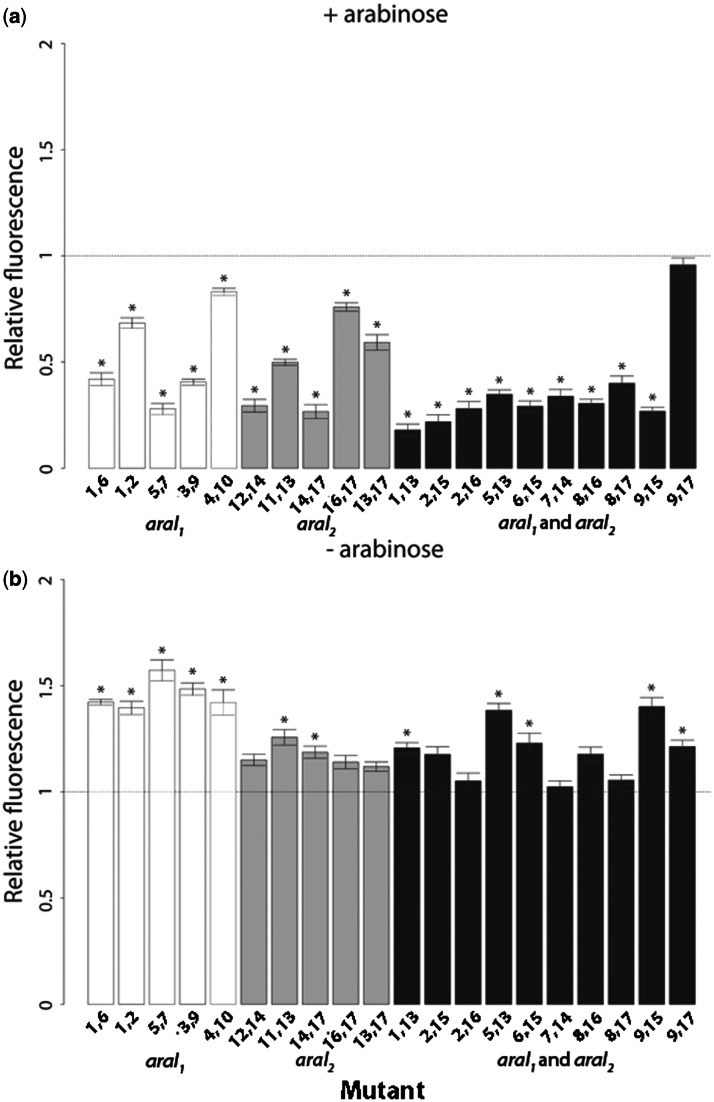
Relative fluorescence of double mutants in two environments. Bars are mean fluorescence relative to wild type. White bars are double mutants with both mutations in the operator *araI*_1_; light gray bars with both mutations in *araI_2_*; and dark bars have one mutation in each of the regions (*araI_1_* and *araI_2_*). Stars indicate mutants that significantly differ from the wild type. The dotted line represents the wild-type relative fluorescence of 1. Error bars are standard errors of the mean. Measurements were taken in the (*a*) presence and (*b*) absence of arabinose.

Most mutations, both single and double, significantly altered expression relative to the wild type (figs. 2 and 3 and supplementary tables S1 and S2, Supplementary Material online), which is not surprising as all mutated sites are fully conserved within the Enterobacteriaceae family (supplementary fig. S2*c*, Supplementary Material online). Surprisingly, three mutations increased expression in the presence of arabinose (fig. 2*a*), in apparent contradiction to previous reports that all possible mutations in tested sites decreased AraC binding by at least 10-fold (Niland et al. 1996). We tested if this disparity arose from the fact that the construct was on a low copy number plasmid rather than on the chromosome, but found no difference in the response of the two systems to arabinose (supplementary fig. S1, Supplementary Material online). The observed results, however, are in accordance with the position weight matrix for AraC binding (supplementary fig. S2, Supplementary Material online) obtained from RegulonDB (Salgado et al. 2013). As these sites are fully conserved, our result suggests that the optimal level of *araBAD* expression, even in the presence of arabinose, is lower than the maximum possible expression level. In the absence of arabinose, all tested mutations either significantly increased expression from *araBAD* CRE, leading to less tight repression, or left it unaffected (figs. 2*b* and 3*b* and supplementary table S2, Supplementary Material online).

### Epistatic Interactions Are Environment Dependent

**Fig. 4.**
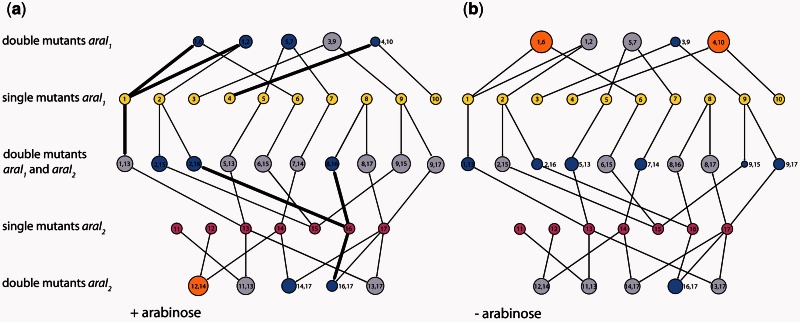
Epistasis in the mutation network. Single mutations are shown in yellow and purple, and are connected to the corresponding double mutant(s) that contain them. For double mutants, the size of the circle is proportional to the magnitude of epistasis, with more negative epistasis values corresponding to smaller circles. Significant negative epistasis is shown in blue, and significant positive interactions in orange, while noninteracting mutations are in gray. Thick connecting lines indicate sign epistasis, with the effect of a single mutation having an opposite sign to that of a double mutant. Interaction network was measured in (*a*) the presence of arabinose and (*b*) the absence of arabinose.

We identified significant epistatic interactions, measured as the deviation of the double mutant expression from the multiplicative expectation of expression based on the corresponding single mutant effects, for exactly half (10 out of 20) of the double mutants in both environments (fig. 4 and supplementary tables S3 and S4, Supplementary Material online). Negative epistasis, where the expression of a double mutant is less than expected, was predominant in both environments (fig. 4): Only one double mutant in the presence of arabinose and two in its absence exhibited positive epistasis (fig. 4). In the presence of arabinose, sign epistasis was observed in 6 of the 10 epistatically interacting double mutants, as one of the component single mutations individually had a positive effect on expression while the double mutant negatively affected transcription (fig. 4*b*). The presence of epistasis did not depend on the magnitude of double mutant effects in either environment (supplementary fig. S4, Supplementary Material online).

Interestingly, these epistatic interactions were environment dependent, as evidenced by a significant G × G × E interaction (*F*_19,120_ = 21.51, *P* < 0.0001). This interaction was not dependent on the three double mutants that changed the sign of interaction between two environments. The identities of most epistatically interacting double mutants differed depending on the presence of arabinose, sometimes even changing the sign of interaction between the two environments (fig. 4).

### Epistatic Interactions Depend on the Physical Location of Mutations

**Fig. 5.**
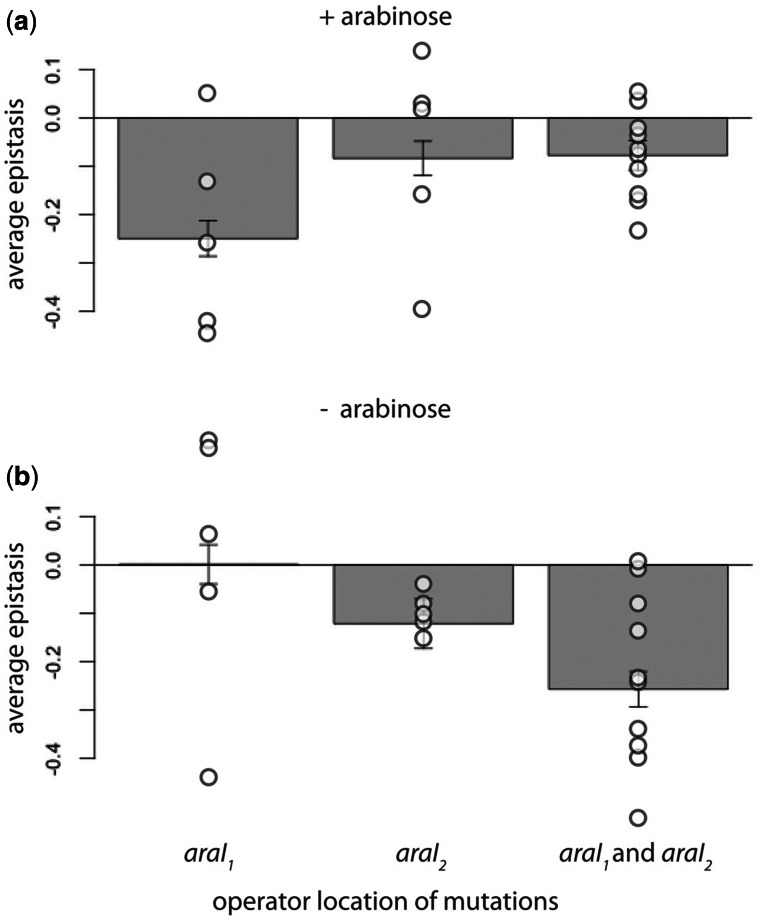
Epistatic interactions depend on the location of mutations. Bars are mean epistasis values for double mutants in *araI_1_*, in *araI_2_*, and those with one mutation in each of the regions (*araI_1_* and *araI_2_*), respectively. Circles are epistasis values for individual double mutants measured in the (*a*) presence of arabinose and (*b*) absence of arabinose. Error bars are standard deviations.

We asked whether epistatic interactions differ depending on the location of the mutations—whether the magnitude and sign of epistasis differ if the mutations are in the *araI_1_*, *araI_2_*, or with one mutation in each of the operators. We found a significant effect of which operator the mutations were in, both in the presence (*F*_2,17_ = 25.083, *P* < 0.0001) and absence of arabinose (*F*_2,17_ = 39.089, *P* < 0.0001) (fig. 5). We conducted pair-wise tests in order to analyze the differences in epistasis between mutations in the same or in different operators. In the presence of arabinose, we found greater negative epistasis when both mutations were in *araI_1_* than in double mutants in *araI_2_* (*t*_8_ = −3.257, *P* < 0.05), and those with a mutation in each of the two operators (*t*_13_ = −4.304, *P* < 0.001) (fig. 5*a*). In the absence of arabinose, greater negative epistasis was observed in double mutants with mutations in different operators, when compared with those with both mutations in either operator *araI_1_* (*t*_13_ = 4.366, *P* < 0.001) or operator *araI_2_* (*t*_13_ = 2.165, *P* < 0.05).

## Discussion

In this study, we evaluated the epistatic interactions between mutations in a *cis*-regulatory region of the *araBAD* operon. We used a synthetic system that decouples the control of transcription from any posttranslational effects on fitness, thus allowing us an estimate of the effects of epistatic interactions on gene expression. We demonstrated that epistatic interactions are a common feature of the *araBAD* CRE, as we observed them in exactly half of the tested double mutants in both environments (fig. 4). Furthermore, we showed that interactions between mutations are strongly environmentally dependent (fig. 4).

The dependence of epistasis on the environment has been previously reported (You and Yin 2002; Hayden and Wagner 2012; Lalic and Elena 2012; Wang, Diaz Arenas, et al. 2012; de Vos et al. 2013; Flynn et al. 2013; Caudle et al. 2014), but only in proteins and under conditions when the environment elicits a complex cellular response. Here, the sole difference between the two environments is the presence or absence of arabinose, which binds to AraC to form the AraC–arabinose complex (Schleif 2010), which in turn affects the binding affinity of AraC for its operator sites (Lobell and Schleif 2005). We demonstrated that such a simple and controllable environmental change is sufficient to drastically alter epistatic interactions between mutations in operator sites.

The relationship between epistasis and environment plays a crucial role in determining how a population responds to environmental change (de Vos et al. 2013). When epistatic interactions between the same mutations are environment dependent, beneficial genotypes do not necessarily correlate between environments (Coyne et al. 1997, 2000; Goodnight and Wade 2000). In terms of transcriptional regulation, selection toward one optimum in one environment might perturb regulation in the other environment in an unpredictable and uncorrelated manner. To illustrate this point, consider a scenario with strong selection to lower expression when the system is in its activated state (i.e., when arabinose is present). In order to most drastically decrease expression, selection might favor negatively epistatic mutations, for example, double mutant (4,10) in our experiment. When arabinose becomes depleted from the environment, these same mutations would be in positive epistasis, so that repression is less tight and efficient. Such effects would be particularly emphasized when mutations exhibit sign epistasis in one environment but not in the other, and therefore a more rugged fitness landscape is translated into a smoother one. Given the frequency and relative importance of changes in CREs for evolution (Wray 2007), understanding how fitness landscapes change between environments is of critical importance.

We also demonstrated that epistasis depends on the physical location of mutations, and in particular whether mutations are located in the same or in different operators (fig. 5). This phenomenon is conceptually similar to the prediction that epistasis is stronger when mutations are found in the same genes (Szendro et al. 2013). In the presence of arabinose, when the system is in its activated state, the mutations in operator *araI_1_* show stronger negative epistasis than those in operator *araI_2_* (fig. 5*a*). This might reflect the stronger binding affinity of the AraC–arabinose dimer for *araI_1_* than for *araI_2_* (Niland et al. 1996). In the absence of arabinose, the *araI_1_* operator plays a dominant role in repression, as the AraC dimer is bound to it and to operator *araO_2_* to form a DNA loop (Lobell and Schleif 2005). Given this model of AraC regulation, the observation of any effects of single mutations in *araI_2_* on expression as well as the occurrence of epistatic interactions between them in the absence of arabinose is surprising, as *araI_2_* should not be bound to AraC (Schleif 2010). It is possible that mutations in operator *araI_2_* affect the accessibility of *araI_1_* by modifying the local tertiary structure of the DNA, as has been shown for mutations at sites flanking the transcription factor binding site (Levo and Segal 2014). Such effects depend on the local DNA context (Gordân et al. 2013), so that changes in the stability of the DNA loop could alter mutational effects on expression. Therefore, a mutation in operator *araI_2_* that increases expression in the absence of arabinose by destabilizing the looping structure could be doing so by modifying the local DNA structure and hence decreasing AraC binding at operator *araI_1_.* Such an effect could also explain the observed negative epistasis when the two mutations are in different operators, as they are not predicted to directly interact (fig. 5*b*).

In this study, we explored the effects of mutations only on the direct phenotype, that is, gene expression, allowing measurements of epistasis present in the genotype–phenotype map. Epistatic interactions can also arise from nonlinearity in the phenotype-fitness mapping (de Visser et al. 2011). By creating an isolated, synthetic system we studied only the nature of the genotype–phenotype mapping and its environment dependence, without considering any downstream effects on fitness. As fitness effects of mutations on CREs depend on the particular properties of the regulated gene, and cannot be a priori inferred (Wittkopp and Kalay 2012), constructing a full genotype–phenotype fitness map was beyond the scope of this work.

Our observation of the dependency of epistatic interactions on the environment and the location of mutations have implications not only for the evolution of CREs but also for engineering regulatory elements with desired expression profiles, a critical task for the optimal design of functional synthetic systems (Purnick and Weiss 2009; Wang, Ma, et al. 2012; Levo and Segal 2014). Epistatic interactions might also impact the overall function of a synthetic construct, potentially leading to unpredictable network properties (Guet et al. 2002). We have shown that large modifications to expression levels are more likely to be achieved by modifying the operator site with highest affinity for the transcription factor, as the epistatic interactions in that operator might be stronger. The fine tuning of expression, on the other hand, is best achieved by introducing mutations in different operators. Thus, the epistatic landscape of CREs may have arisen from the difficulty in finding solutions to two opposing forces—optimal expression in the presence of the inducer and the ability to repress the operon when not required.

## Materials and Methods

The transcription factor, AraC, has five operator binding sites in the CRE of the *araBAD* operon—two proximal sites (*araI_2_* and *araI_1_*), two intermediate sites (*araO_1L_* and *araO_1R_*), and a distal site (*araO_2_*) (Schleif 2010). In the absence of the natural inducer arabinose, AraC exists predominantly as a dimer bound to the *araI_1_* and *araO_2_* operator sites, forming a DNA loop that prevents transcription (Schleif 2003) (fig. 1*a*). The sugar arabinose acts as an inducer, by binding to AraC and introducing a conformational change that prevents DNA looping by the preferential binding of the AraC–arabinose complex to the *araI_1_* and *araI_2_* sites (fig. 1*b*). In addition, binding at the *araI_2_* site directly recruits RNAP, activating transcription of the operon (Schleif 2010).

### Construction of the Plasmid System

The experimental construct consisted of the native *E. coli* K-12 *araBAD* operon regulatory region containing the promoter (*P*_BAD_), the upstream regulatory region consisting of five AraC operators involved in *araBAD* operon regulation (*araI_1_*, *araI_2_*, *araO_1L_*_,_ *araO_1R_*, and *araO_2_*), and the *araC* gene followed by a terminator sequence (fig. 1*c*). The *araBAD* operon was replaced by the fluorescent protein *venus-YFP* (Nagai et al. 2002), followed by the *E. coli* alpha operon tL17 terminator. This construct allows the effects of mutations in the *araBAD* operators to be measured in terms of their effect on expression. The whole construct was cloned into a low copy number plasmid pZS* with a kanamycin resistance marker (Lutz and Bujard 1997).

### Mutant Library Construction

A library of single and double mutants in the *araI_1_* and *araI_2_* operators involved in the regulation of the *araBAD* promoter was created using Quick-Change II^tm^ site-directed mutagenesis protocol (Agilent Technologies). Following the mutagenesis protocol, plasmids were cloned into BW25113 strain (CGSC# 7636) in which the *araBAD* operon has been deleted (Datsenko and Wanner 2000), and were then plated on Luria-Bertani (LB) plates and 50 µg/ml kanamycin.

We introduced point mutations in all base pairs in *araI_1_* and *araI_2_* operators for which it was previously reported that any substitution decreased AraC binding by at least 10-fold (Niland et al. 1996). Based on a search of public sequence databases, the *araI_1_* and *araI_2_* operator sites are highly conserved within the Enterobacteriaceae family (supplementary fig. S2*c*, Supplementary Material online). For each mutation, we randomly selected the base to be introduced, the only constraint being that the ratio between transitions and transversions was approximately 1:2. We tested a total of 17 single mutants, 10 in *araI_1_* and 7 in *araI_2_* (fig. 1), and 20 double mutants. The double mutations consisted of random combinations of single mutants. For five of the double mutants, both mutations were in the *araI_1_* operator; five were both in the *araI_2_* operator; and ten had one mutation in each of the two operators (supplementary fig. S4, Supplementary Material online).

### Expression Assays

Single colonies with the desired point mutations (each confirmed by Sanger sequencing) were grown overnight at 37 °C on LB containing 50 µg/ml kanamycin. These cultures were used to inoculate four replicate populations in arabinose and four in the absence of arabinose. These were grown overnight in M9 media, supplemented with 0.01% casamino acids, 50 µg/ml kanamycin, 0.2% glycerol, and either containing 0.1% arabinose or not. The populations were then serially diluted, grown for 4 h, and then used to inoculate 1.2 ml of the corresponding media with 0.1% of the grown culture, to ensure that measurements are taken during the exponential growth. When the cultures reached an OD_600_ of approximately 0.05, 150 μl of the culture was sampled and fluorescence and OD_600_ measurements taken using Biotek H1 plate reader. Fluorescence measurements were normalized by the OD_600_ measurements to account for the variation in the size of the initial inoculum. With this design, the expression of each mutant was measured four times in each environment, with measurements performed on independent cultures.

### Chromosome Insertion

The observed disparity between the effects of mutations measured by Niland et al. (1996) and in our study could have been a result of a difference in how the system behaves when on a low copy number plasmid or in the chromosome. To test if this were true, we inserted the wild-type construct with the kanamycin resistance gene into the *araBAD–araC* locus on the BW25113 chromosome using lambda-red recombineering. We tested how the two systems respond to arabinose by measuring fluorescence in the manner previously described, on a variety of arabinose concentrations (0.2%, 0.1%, 0.05%, 0.025%, 0.0125%, 0.00625%, 0.003125%, and 0%). We used six replicates at each concentration.

### Data Analysis

#### Expression across the Mutant Library

For each mutant, we measured the fluorescence and normalized it by the fluorescence for the wild-type plasmid in the same environment. Because there is variation in the fluorescence of the wild-type strain, we used error propagation when calculating standard deviations of the mean normalized fluorescence (Ku 1966). We tested for an effect of each mutant on expression using ANOVA (aov function in R statistical software version 3.1.1.; R Core Team 2015), with relative fluorescence as the response variable, mutation as the fixed factor (37 levels), and replicate (4 levels) as a random factor, and used post hoc FDR-corrected *t*-tests to compare the mean fluorescence of each mutant to that of the wild type.

#### Epistatic Interactions

To estimate the interaction between two mutations, we used a multiplicative epistasis model, as the studied mutations were not expected to be independent of each other (Cordell 2002). In this model, epistasis is calculated as ε = ω_m12_ − ω_m1_ × ω_m2_, where ω_m12_ is the relative fluorescence of a double mutant, and ω_m1_ and ω_m2_ the relative fluorescence of the two corresponding single mutants, respectively. It is worth noting that we calculated epistasis based on expression levels of a reporter gene, rather than based on the strength of binding of the transcription factor to the operator. The relationship between the epistasis based on expression to that based on binding depends on the role of the transcription factor and the context of its binding. Broadly speaking, if two mutations have lower expression than expected based on the single mutant phenotypes (i.e., show negative epistasis on expression), this might be due to negative epistasis on activator binding, or positive epistasis on repressor binding.

To test whether the estimated epistasis was significantly different from zero, we conducted a series of FDR-corrected *t*-tests. The errors were calculated based on four replicates, using error propagation to account for the inherent variance of each replicate that was due to normalization by the wild type. To test for a relationship between epistasis and the magnitude of the corresponding double mutant effects, we used a linear regression model weighted by the cumulative error. To test for the effects of the environment on epistasis, we tested for a genotype-by-genotype-by-environment (G × G × E) interaction using ANOVA, with replicate as a random factor. The difference in the magnitude and sign of epistasis was compared between the double mutants depending on the region that the mutations were in (both mutations in operator *araI_1_*, both in operator *araI_2_*, or one mutation in each of the two operators). FDR-corrected pairwise *t*-tests were carried out between the three regions, with mean epistasis as a response variable. We tested whether magnitude of epistasis depended on the proximity of mutations within an operon by carrying out ANOVA with distance between introduced mutations in base pairs as the fixed factor. Possibly due to small sample size, we did not find a significant effect of the distance between mutations in either environment (in the absence of arabinose: *F*_1,8_ = 0.206, *P* = 0.662; in the presence of arabinose: *F*_1,8_ = 0.163, *P* = 0.697).

## Supplementary Material

Supplementary figures S1–S4 and tables S1–S4 are available at *Molecular Biology and Evolution* online (http://www.mbe.oxfordjournals.org/).

Supplementary Data
