# Supplementary material for: Epistatic Interactions in the Arabinose Cis-Regulatory Element
Source: Mol Biol Evol. 2015 Nov 20;33(3):761–9. doi: 10.1093/molbev/msv269 (PMC4760080; doi:10.1093/molbev/msv269)
Supplement: Supplementary Data [file supp_33_3_761__index.html]

Epistatic Interactions in the Arabinose Cis-Regulatory Element — Epistatic Interactions in the Arabinose Cis-Regulatory Element — Supplementary Data 

# Epistatic Interactions in the Arabinose *Cis*-Regulatory Element

## Supplementary Data

files

- Supplementary Data - docx file
