# Supplementary material for: Epistatic Interactions in the Arabinose Cis-Regulatory Element
Source: Mol Biol Evol. 2015 Nov 20;33(3):761–9. doi: 10.1093/molbev/msv269 (PMC4760080; doi:10.1093/molbev/msv269)
Supplement: Supplementary Data [file supp_msv269_MLagator_Supplementary_Materials.docx]

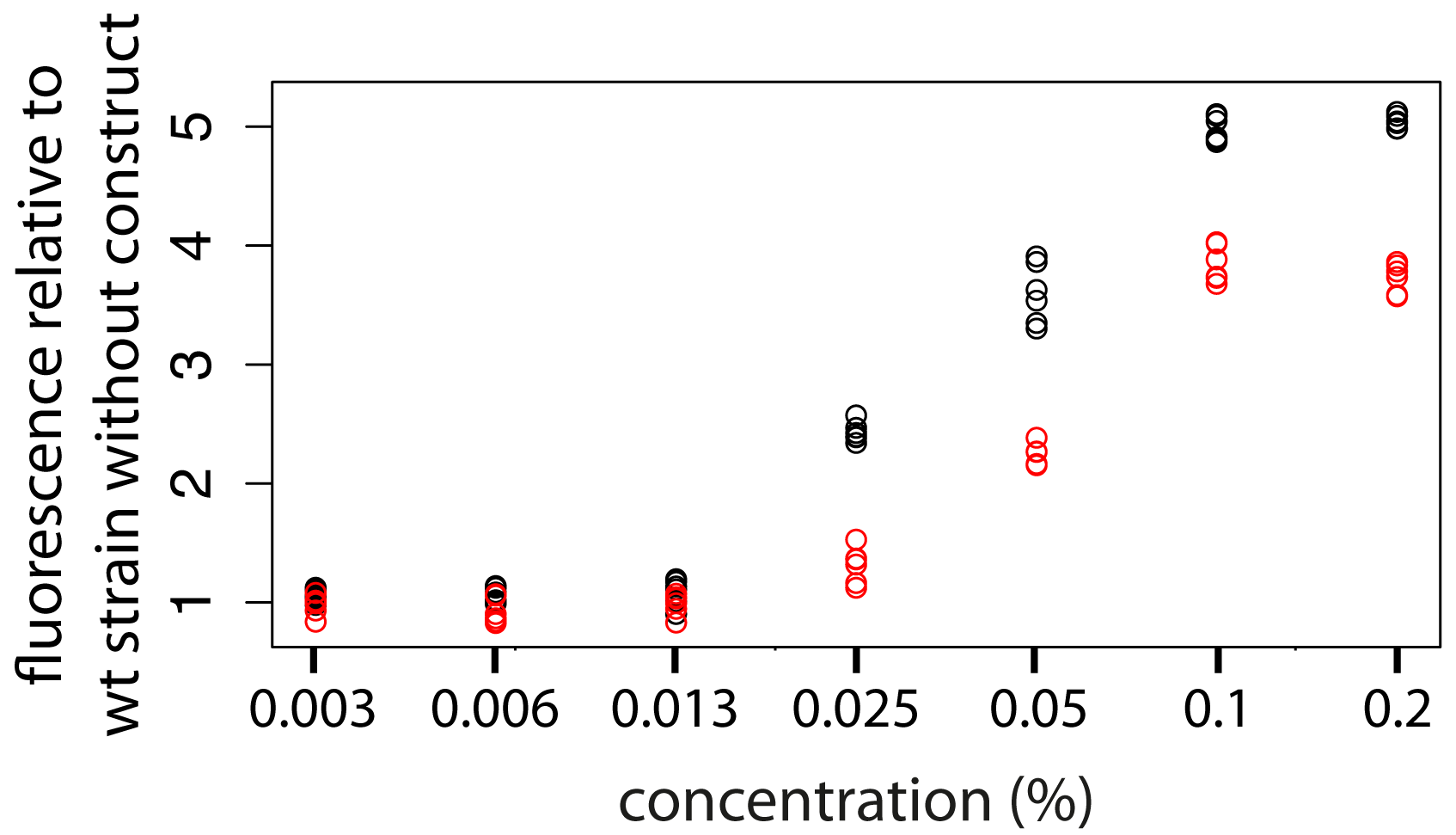


**Figure S1.** Relative fluorescence as a function of arabinose concentration in the wild type construct on the plasmid (black) and on the chromosome (red). Each point represents relative fluorescence of one of six replicates measured at a given arabinose concentration.


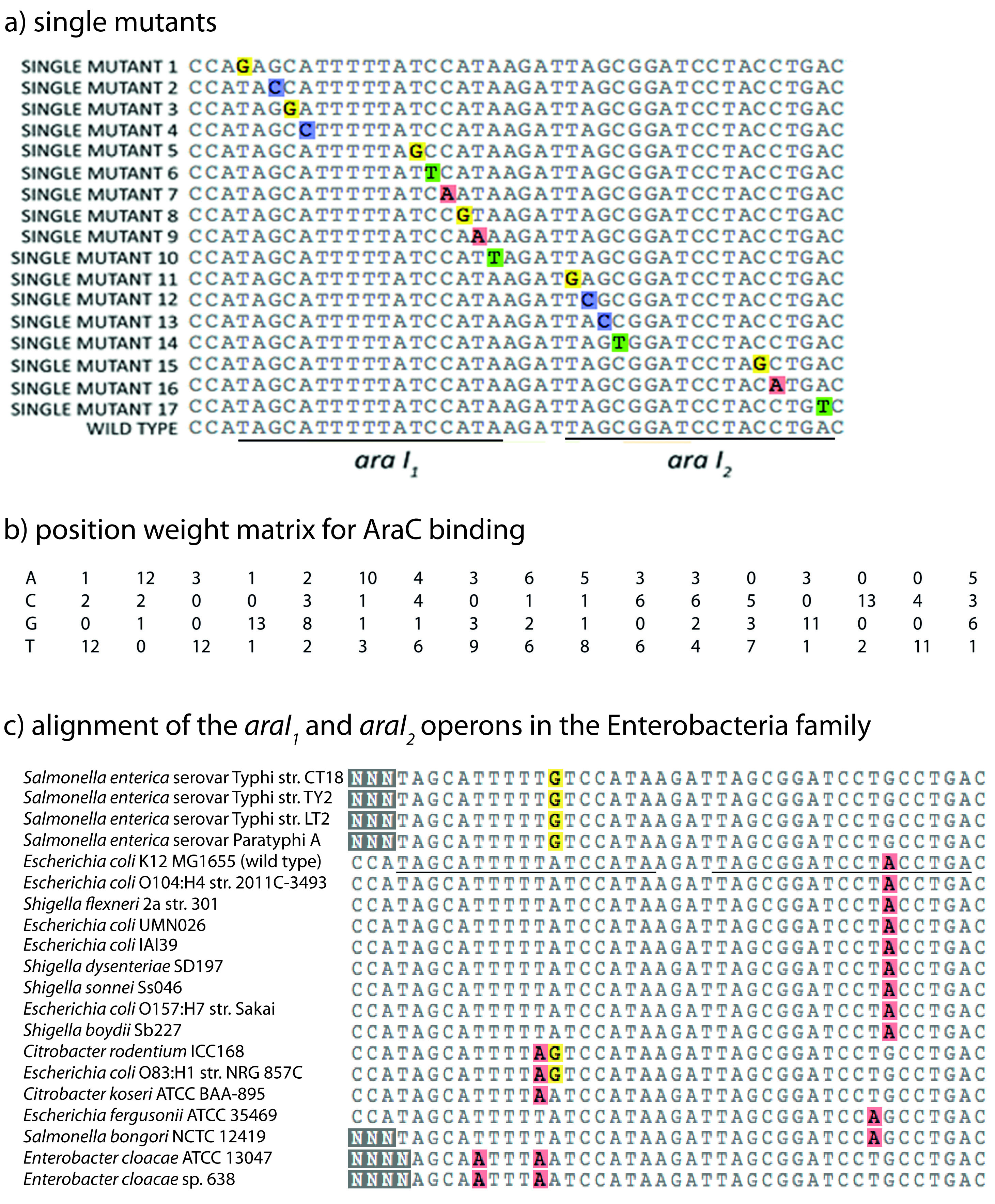


**Figure S2.** Single mutant library. Ten single mutants in *araI_1_* (1-10), and seven in *araI_2_* region (11-17). b) Position weight matrix for AraC binding to its operator site in the absence of arabinose, obtained from RegulonDB. Higher numbers indicate higher binding affinity. c) Alignment of two *araC* operons across the Enterobacteriae family, showing no divergence in the mutated sites.


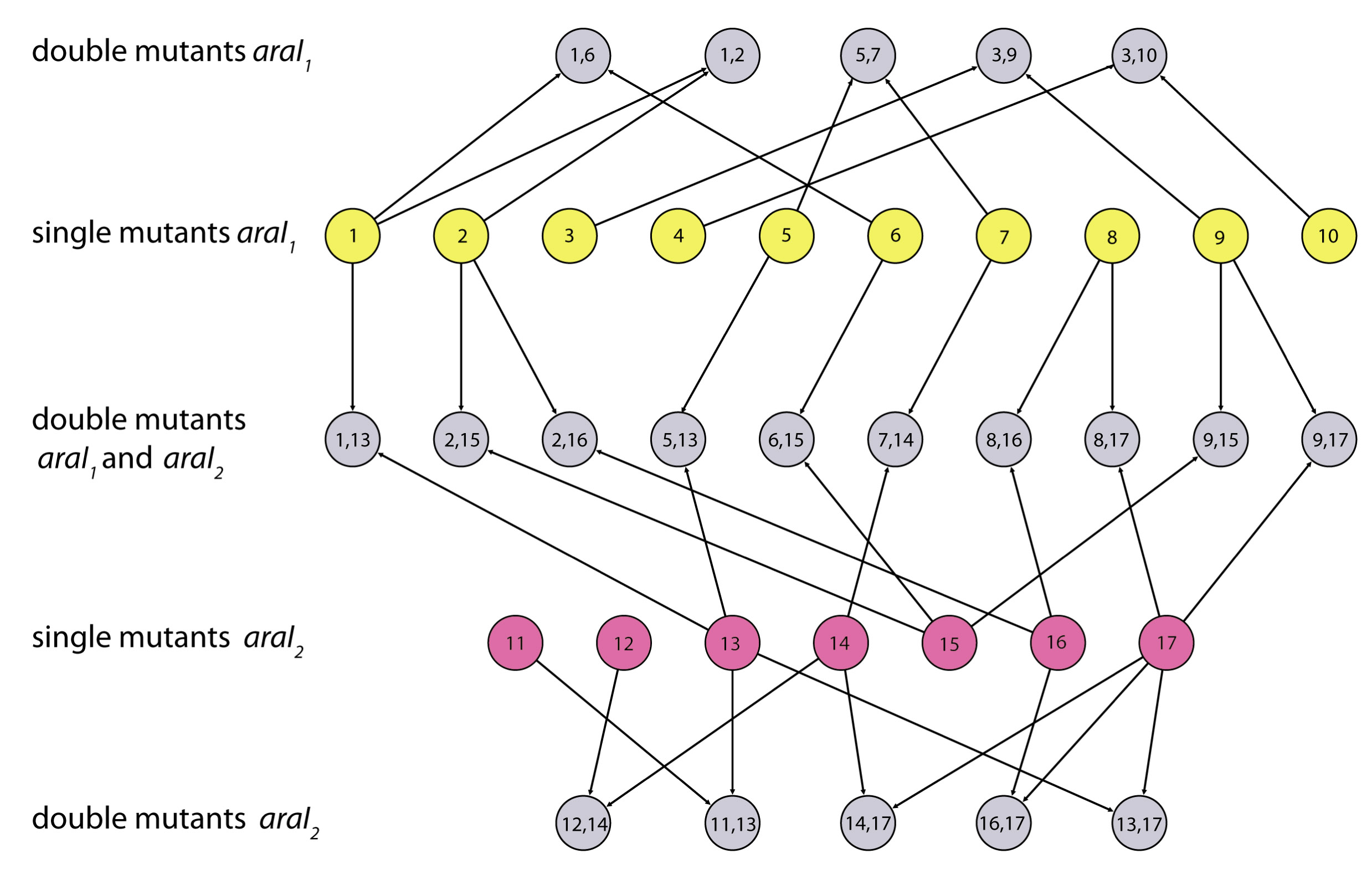


**Figure S3**. Network of mutants used in the experiment. Single mutants are shown in yellow and purple, and are connected to the corresponding double mutant(s) that contains them. The numbering of single mutants corresponds to that in Fig.S1.

**Table S1**. fdr-corrected t-tests comparing the relative fluorescence of each of the mutants to the wild type in the presence of arabinose.

**+Arabinose** Estimate Std. Error t value Pr(>|t|)

(Intercept) 15805.2 504.7 31.316 <0.001 ***

mutantm1 2543.6 713.8 3.564 0.0136 *

mutantm2 -2960.7 713.8 -4.148 <0.01 **

mutantm3 -3609.2 713.8 -5.057 <0.01 ***

mutantm4 6523.0 713.8 9.139 <0.01 ***

mutantm5 -4692.5 713.8 -6.574 <0.01 ***

mutantm6 -4355.4 713.8 -6.102 <0.01 ***

mutantm7 -6493.5 713.8 -9.098 <0.01 ***

mutantm8 -10802.1 713.8 -15.134 <0.01 ***

mutantm9 -8425.7 713.8 -11.805 <0.01 ***

mutantm10 -1297.1 713.8 -1.817 0.6734

mutantm11 -4869.8 713.8 -6.823 <0.01 ***

mutantm12 -9484.8 713.8 -13.289 <0.01 ***

mutantm13 -10024.9 713.8 -14.045 <0.01 ***

mutantm14 -1267.6 713.8 -1.776 0.7075

mutantm15 -8510.6 713.8 -11.924 <0.01 ***

mutantm16 5885.8 713.8 8.246 <0.01 ***

mutantm17 -2777.6 713.8 -3.891 <0.01 **

mutantm1,6 -9181.2 713.8 -12.863 <0.01 ***

mutantm1,2 -5002.0 713.8 -7.008 <0.01 ***

mutantm5,7 -11391.3 713.8 -15.960 <0.01 ***

mutantm3,9 -9390.5 713.8 -13.156 <0.01 ***

mutantm4,10 -2690.7 713.8 -3.770 <0.01 **

mutantm12,14 -7924.5 713.8 -11.102 <0.01 ***

mutantm11,13 -11589.6 713.8 -16.237 <0.01 ***

mutantm14,17 -6441.6 713.8 -9.025 <0.01 ***

mutantm16,17 -3805.8 713.8 -5.332 <0.01 ***

mutantm13,17 -11151.6 713.8 -15.624 <0.01 ***

mutantm1,13 -10447.9 713.8 -14.638 <0.01 ***

mutantm2,15 -11571.1 713.8 -16.211 <0.01 ***

mutantm2,16 -679.3 713.8 -0.952 0.9997

mutantm5,13 -12961.2 713.8 -18.159 <0.01 ***

mutantm6,15 -11195.2 713.8 -15.685 <0.01 ***

mutantm7,14 -9487.8 713.8 -13.293 <0.01 ***

mutantm8,16 -12350.8 713.8 -17.304 <0.01 ***

mutantm8,17 -10978.2 713.8 -15.381 <0.01 ***

mutantm9,15 -11388.1 713.8 -15.955 <0.01 ***

mutantm9,17 -10312.3 713.8 -14.448 <0.01 ***

**Table S2**. fdr-corrected t-tests comparing the relative fluorescence of each of the mutants to the wild type in the absence of arabinose.

**-Arabinose** Estimate Std. Error t value Pr(>|t|)

(Intercept) 1678.695 70.429 23.835 <0.01 ***

mutantm1 180.603 99.602 1.813 0.6774

mutantm2 570.699 99.602 5.730 <0.01 ***

mutantm3 414.160 99.602 4.158 <0.01 **

mutantm4 3.334 99.602 0.033 1.0000

mutantm5 442.123 99.602 4.439 <0.01 ***

mutantm6 94.685 99.602 0.951 0.9997

mutantm7 348.781 99.602 3.502 0.0167 *

mutantm8 122.417 99.602 1.229 0.9855

mutantm9 1082.899 99.602 10.872 <0.01 ***

mutantm10 372.582 99.602 3.741 <0.01 **

mutantm11 221.046 99.602 2.219 0.3723

mutantm12 344.808 99.602 3.462 0.0188 *

mutantm13 236.200 99.602 2.371 0.2814

mutantm14 238.065 99.602 2.390 0.2718

mutantm15 261.124 99.602 2.622 0.1686

mutantm16 313.208 99.602 3.145 0.0463 *

mutantm17 151.596 99.602 1.522 0.8848

mutantm1,6 709.363 99.602 7.122 <0.01 ***

mutantm1,2 664.307 99.602 6.670 <0.01 ***

mutantm5,7 960.242 99.602 9.641 <0.01 ***

mutantm3,9 223.901 99.602 2.248 0.3530

mutantm4,10 706.251 99.602 7.091 <0.01 ***

mutantm12,14 430.760 99.602 4.325 <0.01 **

mutantm11,13 312.891 99.602 3.141 0.0474 *

mutantm14,17 199.188 99.602 2.000 0.5301

mutantm16,17 234.679 99.602 2.356 0.2889

mutantm13,17 252.702 99.602 2.537 0.2028

mutantm1,13 40.364 99.602 0.405 1.0000

mutantm2,15 672.441 99.602 6.751 <0.01 ***

mutantm2,16 356.489 99.602 3.579 0.0134 *

mutantm5,13 346.639 99.602 3.480 0.0178 *

mutantm6,15 384.236 99.602 3.858 <0.01 **

mutantm7,14 92.105 99.602 0.925 0.9998

mutantm8,16 296.359 99.602 2.975 0.0731 .

mutantm8,17 297.505 99.602 2.987 0.0709 .

mutantm9,15 85.440 99.602 0.858 1.0000

mutantm9,17 642.851 99.602 6.454 <0.01 ***


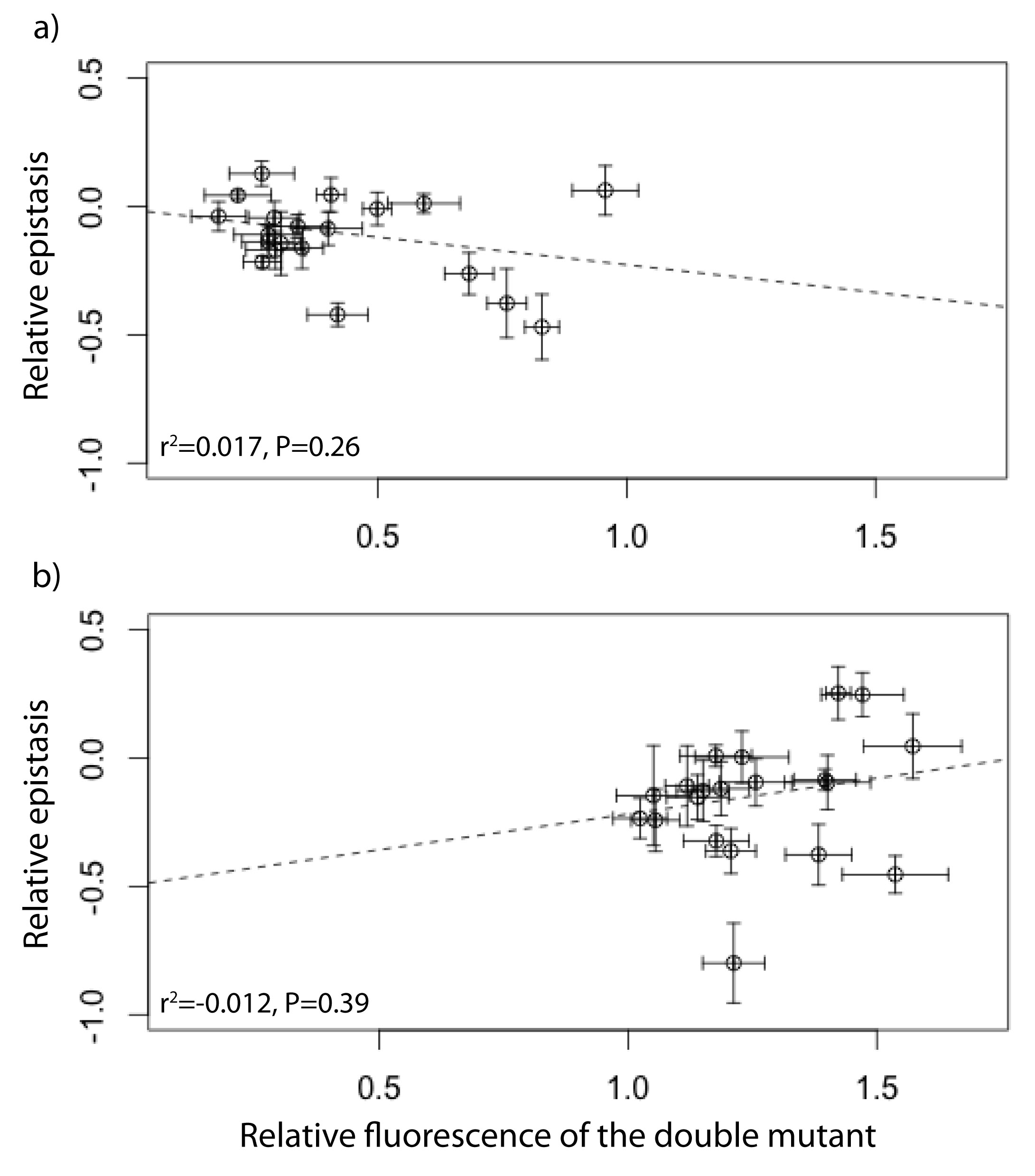


**Figure S4.** Correlation between the effects of double mutations on expression and epistasis in a) the presence of arabinose; and b) the absence of arabinose. Points are mean values for relative fluorescence and epistasis; bars are standard deviations. The broken line is the linear regression.

**Table S3**. Epistasis in the presence of arabinose. The sign of epistasis is the sign of mean epistasis value. The sign of 1^st^ or 2^nd^ mutation indicates whether the effect of that mutation is in the same (magnitude) or the opposite direction (sign) to the effect of the double mutant. P values are fdr-corrected, and red if <0.05.

| double mutant | mean epistasis | epistasis stdev | t value | p value | sign of 1^st^ mutation | sign of 2^nd^ mutation |
| --- | --- | --- | --- | --- | --- | --- |
| 20 | -0,422 | 0,045 | -12,113 | <0,0001 | sign | magnitude |
| 21 | -0,262 | 0,082 | -5,396 | 0,008 | sign | magnitude |
| 22 | -0,138 | 0,064 | -3,312 | 0,032 | magnitude | magnitude |
| 23 | 0,045 | 0,066 | 1,057 | 0,390 | magnitude | magnitude |
| 24 | -0,470 | 0,127 | -6,827 | 0,003 | sign | magnitude |
| 25 | 0,128 | 0,048 | 3,558 | 0,032 | magnitude | magnitude |
| 26 | 0,011 | 0,038 | 0,351 | 0,776 | magnitude | magnitude |
| 27 | -0,170 | 0,073 | -3,768 | 0,031 | magnitude | magnitude |
| 28 | -0,377 | 0,134 | -5,229 | 0,008 | sign | magnitude |
| 29 | -0,009 | 0,064 | -0,228 | 0,827 | magnitude | magnitude |
| 30 | -0,086 | 0,065 | -2,059 | 0,130 | sign | magnitude |
| 31 | -0,110 | 0,039 | -3,348 | 0,032 | magnitude | magnitude |
| 32 | -0,162 | 0,081 | -3,358 | 0,032 | magnitude | sign |
| 33 | -0,078 | 0,047 | -2,213 | 0,125 | magnitude | magnitude |
| 34 | -0,044 | 0,063 | -1,070 | 0,390 | magnitude | magnitude |
| 35 | -0,144 | 0,123 | -2,153 | 0,125 | magnitude | magnitude |
| 36 | -0,217 | 0,025 | -7,446 | 0,003 | magnitude | sign |
| 37 | 0,043 | 0,015 | 1,566 | 0,241 | magnitude | magnitude |
| 38 | 0,062 | 0,096 | 1,137 | 0,390 | magnitude | magnitude |
| 39 | -0,039 | 0,057 | -1,000 | 0,395 | magnitude | magnitude |

**Table S4**. Epistasis in the absence of arabinose. The sign of epistasis is the sign of mean epistasis value. The sign of 1^st^ or 2^nd^ mutation indicates whether the effect of that mutation is in the same (magnitude) or the opposite direction (sign) to the effect of the double mutant. P values are fdr-corrected, and in red if <0.05.

| double mutant | mean epistasis | epistasis stdev | t value | p value | sign of 1st mutation | sign of 2nd mutation |
| --- | --- | --- | --- | --- | --- | --- |
| 20 | 0,253 | 0,103 | 4,896 | 0,007 | magnitude | magnitude |
| 21 | -0,085 | 0,041 | -4,120 | 0,138 | magnitude | magnitude |
| 22 | 0,046 | 0,126 | 0,728 | 0,549 | magnitude | magnitude |
| 23 | -0,454 | 0,073 | -12,484 | 0,000 | magnitude | magnitude |
| 24 | 0,246 | 0,086 | 5,737 | 0,003 | magnitude | magnitude |
| 25 | -0,119 | 0,105 | -2,273 | 0,106 | magnitude | magnitude |
| 26 | -0,109 | 0,156 | -1,391 | 0,251 | magnitude | magnitude |
| 27 | -0,128 | 0,118 | -2,161 | 0,114 | magnitude | magnitude |
| 28 | -0,153 | 0,087 | -3,507 | 0,023 | magnitude | magnitude |
| 29 | -0,095 | 0,092 | -2,050 | 0,123 | magnitude | magnitude |
| 30 | -0,242 | 0,122 | -3,979 | 0,015 | magnitude | magnitude |
| 31 | -0,146 | 0,194 | -1,504 | 0,229 | magnitude | magnitude |
| 32 | -0,377 | 0,118 | -6,366 | 0,003 | magnitude | magnitude |
| 33 | -0,235 | 0,078 | -6,001 | 0,003 | magnitude | magnitude |
| 34 | 0,003 | 0,101 | 0,066 | 0,949 | magnitude | magnitude |
| 35 | -0,323 | 0,061 | -10,534 | 0,000 | magnitude | magnitude |
| 36 | -0,095 | 0,106 | -1,791 | 0,165 | magnitude | magnitude |
| 37 | 0,009 | 0,042 | 0,407 | 0,734 | magnitude | magnitude |
| 38 | -0,799 | 0,156 | -10,268 | 0,000 | magnitude | magnitude |
| 39 | -0,362 | 0,088 | -8,262 | 0,001 | magnitude | magnitude |
